# Public acceptability of nudging and taxing to reduce consumption of alcohol, tobacco, and food: A population-based survey experiment

**DOI:** 10.1016/j.socscimed.2019.112395

**Published:** 2019-09

**Authors:** J.P. Reynolds, S. Archer, M. Pilling, M. Kenny, G.J. Hollands, T.M. Marteau

**Affiliations:** University of Cambridge, UK

**Keywords:** England, Choice architecture, Tax, Smoking, Drinking, Obesity, Communication, Attitudes

## Abstract

There is growing evidence for the effectiveness of choice architecture or ‘nudge’ interventions to change a range of behaviours including the consumption of alcohol, tobacco and food. Public acceptability is key to implementing these and other interventions. However, few studies have assessed public acceptability of these interventions, including the extent to which acceptability varies with the type of intervention, the target behaviour and with evidence of intervention effectiveness. These were assessed in an online study using a between-participants full factorial design with three factors: Policy (availability *vs* size *vs* labelling *vs* tax) x Behaviour (alcohol consumption *vs* tobacco use *vs* high-calorie snack food consumption) x Evidence communication (no message *vs* assertion of policy effectiveness *vs* assertion and quantification of policy effectiveness [e.g., a 10% change in behaviour]). Participants (*N* = 7058) were randomly allocated to one of the 36 groups. The primary outcome was acceptability of the policy. Acceptability differed across policy, behaviour and evidence communication (all *p*s < .001). Labelling was the most acceptable policy (supported by 78%) and Availability the least (47%). Tobacco use was the most acceptable behaviour to be targeted by policies (73%) compared with policies targeting Alcohol (55%) and Food (54%). Relative to the control group (60%), asserting evidence of effectiveness increased acceptability (63%); adding a quantification to this assertion did not significantly increase this further (65%). Public acceptability for nudges and taxes to improve population health varies with the behaviour targeted and the type of intervention but is generally favourable. Communicating that these policies are effective can increase support by a small but significant amount, suggesting that highlighting effectiveness could contribute to mobilising public demand for policies. While uncertainty remains about the strength of public support needed, this may help overcome political inertia and enable action on behaviours that damage population and planetary health.

## Highlights

• Public acceptability varies across nudges and taxes and behaviour targeted.
• Labelling was the most acceptable policy, then Size, Tax, and Availability.
• Targeting tobacco use was more acceptable than targeting alcohol or food.
• Asserting effectiveness increased acceptability; quantifying it had no added impact.
• The more participants engage in the targeted behaviour, the lower their support.

## 1 Introduction

Reducing the consumption of alcohol, tobacco, and unhealthy foods would reduce rates of prevalent non-communicable diseases including type 2 diabetes, many cancers, and heart disease (Lim et al., 2012; Steel et al., 2018; Whiteford et al., 2013). Such behaviour change would also have broader effects by making an impact on the global syndemic of obesity, undernutrition, and climate change (Swinburn et al., 2019). Achieving the necessary scale of behaviour change requires multiple interventions across multiple environments or systems (McGowan et al., 2018). One particularly effective set of interventions for changing these behaviours involves reducing demand via price increases (Marten et al., 2018). Another set of interventions that show promise comprise those targeting cues in physical environments, commonly known as altering the choice architecture, or nudging (Thaler and Sunstein, 2008). These interventions generally involve changing some aspect of physical environments that shape our behaviour often without our awareness - be it the etching of a housefly in a urinal to ‘improve the aim’ or the chevrons painted on a road to create an illusion of speed to slow drivers. Building on a typology for these interventions to change health-related behaviours by altering cues in proximal physical environments (Hollands et al., 2017a,b; Marteau et al., 2012) our focus here is upon such interventions to reduce consumption of alcohol, tobacco and food.

Public acceptability of policies *i.e.* how individuals feel and think about the implementation or continued existence of policies (Sekhon et al., 2017) is increasingly recognised as playing a pivotal role in determining the extent to which evidence is implemented into policy (Cairney, 2009; Cullerton et al., 2016; Freudenberg, 2014; Roache and Gostin, 2018; Smith, 2013). The focus of the current paper is the public acceptability of policies that target the consumption of tobacco, alcohol and food to improve population health.

Public acceptability is generally highest for information-based interventions such as educational campaigns, with 70–90% of the population supporting such policies (Hagmann et al., 2018; Petrescu et al., 2016). These interventions, however, are often less effective than more structural interventions such as taxation, which are typically supported by 40–50% of the population (Diepeveen et al., 2013; Petrescu et al., 2016; Reynolds et al., 2018a). This low support for taxation is explained by a widely held belief that it does not change behaviour and by a mistrust in the government's use of these taxes (Somerville et al., 2015; Thomas-Meyer et al., 2017). The acceptability of nudging tends to be neither as low as for price-based interventions nor as high as for information-based interventions (Sunstein, Reisch and Rauber, 2018b). Examples of these nudges include altering portion sizes of alcohol, tobacco, and food (Hollands et al., 2015), altering the relative availability of healthier food options (Hollands et al., Under review; Pechey et al., 2019) and adding nutritional or warning labels on alcohol, tobacco, and food products (Crockett et al., 2018; Ngo et al., 2018). The evidence for the acceptability of these interventions is currently limited, and uncertainty remains about the extent to which acceptability varies with the type of policy and the target behaviour, as well as the impact on acceptability of how evidence for effectiveness is presented. This study is the first to compare the effect of these three key factors in terms of public attitudes.

The perceived effectiveness of a policy is one of the strongest predictors of its acceptability (Bos et al., 2015; Mazzocchi et al., 2015; Storvoll et al., 2015). Therefore, communicating evidence of a policy's effectiveness has the potential to align perceived effectiveness with acceptability. A recent systematic review of experimental evidence across policy domains that included health, the environment, education and gun control found that communication of evidence of effectiveness increased support for a policy by approximately four percentage points, with communication of evidence of ineffectiveness decreasing support by a similar amount. (Reynolds et al., Under review). It is likely that communicating this evidence of policy effectiveness changed participants' beliefs about the effectiveness of the policy, which in turn increased their support for it (Reynolds et al., 2018a).

There is little empirical research testing the most effective ways of communicating evidence of a policy's effectiveness (Brick et al., 2018). The aforementioned review (Reynolds et al., Under review) found that the most common forms in which evidence was communicated included unquantified assertions of effectiveness*, e.g*. “Getting this law in place is one way to protect the public from dangerous guns” (McGinty et al., 2013) and quantified estimates of effectiveness, e.g. “After the tax is implemented, childhood obesity will drop from 14% to 12.3%” (Reynolds et al., 2018a). Reynolds et al. (Under review) compared these two forms of evidence communication and their impact on acceptability, with no clear evidence favouring either (Reynolds et al., Under review). The extensive literature on the communication of risks, harms and benefits to individuals provides some guidance for communicating similar types of information, albeit about harms and benefits of a policy for a population or group (Brick et al., 2018; Spiegelhalter, 2017). Using descriptive words alone, without quantification, to communicate risk is generally discouraged as words can be ambiguous (Berry and Hochhauser, 2006; Spiegelhalter, 2017). For example, a tax on sugar that is described as ‘very effective’ may lead one person to infer that the tax will reduce sugar consumption by 5%, whereas another may infer this to mean a reduction of 50%. In addition to providing clarity, numbers can also bolster trust in the information (Visschers et al., 2009). Whether these benefits of quantification translate into changes in beliefs and attitudes has yet to be determined.

The aim of the current study is to estimate the public acceptability of policies (nudges and taxes), targeting three sets of behaviour (alcohol consumption, tobacco use, and high-calorie snack consumption) to improve health outcomes. A further aim is to test whether communicating evidence of policy effectiveness (either asserted, or asserted and quantified) increases the public acceptability of these policies across these behaviours.

## 2 Methods

The study was preregistered with the Open Science Framework (DOI: *withheld to ensure anonymity of authors during peer review, see anonymised protocol attached as a supplement*).

### 2.1 Participants

A research agency (www.yougov.co.uk) recruited 7058 participants from their online panel representative of the English population based on age, gender, socioeconomic status, region, and education. This sample size provided 80% power to explore the 3-way interaction term (policy x behaviour x effectiveness communication) with *α* = 0.05 and an effect size of *f* = 0.05. Data were collected between 9th and 16th October 2018.

The research agency provided sample weights that were used in all analyses to ensure the sample was representative of the English population. See Table S1 in the supplement for the demographic characteristics of the sample.

### 2.2 Design

An online study using a between-participants full factorial design with three factors resulting in 36 groups: Policy (availability *vs* size *vs* labelling *vs* tax) x Behaviour (alcohol consumption *vs* tobacco use *vs* high-calorie snack food consumption) x Evidence communication (no message *vs* assertion of policy effectiveness *vs* assertion and quantification of policy effectiveness). Participants were randomly allocated to one of the 36 groups, using the research agency's software.

### 2.3 Interventions

The four policies were: *i.* a ban on the product in local corner shops to reduce availability (Availability); *ii.* a reduction in the size of the product (Size); *iii*. a graphic warning label on the product (Labelling), and *iv*. a tax to increase the price of the product (Tax).

The three behaviours that were targeted were the use or consumption of: *i.* Alcohol, *ii.* Tobacco, and *iii.* Food (high-calorie snack foods). Descriptions of all twelve policies - four for each of the three behaviours - are presented in Box 1.

Box 1 **Descriptions of the 12 policies assessed.**

**Table:** 

	Alcohol	Tobacco	Snacks
Availability	a new policy to ban the sale of alcohol in corner shops	a new policy to ban the sale of cigarettes in corner shops	a new policy to ban the sale of high calorie snacks (e.g. crisps and sweets) in corner shops
Size	a new policy to reduce the serving size of alcoholic drinks in pubs and restaurants	a new policy to reduce the number of cigarettes in a pack	a new policy to reduce the size of packets of high calorie snacks (e.g. crisps and sweets)
Labelling	a new policy to add graphic warning labels to alcohol	a new policy to add graphic warning labels to cigarettes	a new policy to add graphic warning labels to high calorie snacks (e.g. crisps and sweets)
Tax	a new policy to increase the price of alcohol	a new policy to increase the price of cigarettes	a new policy to increase the price of high calorie snacks (e.g. crisps and sweets)

Alt-text: Box 1

The three evidence communication messages were *i.* no message (control group) *ii*. a message that asserted that the policy was effective at changing the targeted behaviour, without any description of the magnitude of the effectiveness, and *iii.* a message that asserted that the policy was effective and quantified the magnitude of the effectiveness.

Within the asserting and quantifying condition, the effectiveness was expressed as a 10% reduction for all behaviours and policies to ensure consistency and comparability across the groups. This size of effect was selected as it is credible across conditions given existing evidence. For example, Cochrane reviews suggest that energy labelling on menus in restaurants could reduce energy purchased per meal by 7.8% (Crockett et al., 2018), that smaller portions, packages and tableware could reduce average daily energy consumed from food by 8.5%–13.5% among UK children and adults (Hollands et al., 2015), and that reducing availability of a range or category of food(s) could reduce its consumption by 17% (Hollands et al., Under review). There is also evidence that implementing a tax on sugar sweetened beverages led to a 9.6% drop in sales (Silver et al., 2017). The wording of policies was also matched across the 36 groups as far as possible to ensure consistency. In one case this led to a policy that was already implemented *i.e.* a graphic warning label on cigarette packs. To address this the wording used for all groups was “… a new graphic warning label …”.

Text provided to three of the 36 groups is presented in Box 2 (See the supplement for the complete set of texts provided to the 36 groups).

Box 2 **Three example intervention messages.**

**Table:** 

Alcohol/Availability/Asserted evidence: “*The government is considering a new policy to ban the sale of alcohol in corner shops to help people drink less. Research shows that the introduction of this new policy will reduce the number of people who drink in ways that harm their health.”* Tobacco/Tax/Asserted and Quantified evidence: “*The government is considering a new policy to increase the price of cigarettes to help people stop smoking. Research shows that the introduction of this new policy will reduce the number of people who smoke by 10%.”* Food/Size/No evidence: *“The government is considering a new policy to reduce the size of packets of high calorie snacks (*e.g. *crisps and sweets) to tackle obesity. The price will be reduced in line with the change in size.*

Alt-text: Box 2

### 2.4 Measures

*Primary outcome:* Acceptability of the policy was assessed using three items (*α* = 0.97) (Reynolds et al., 2018a): “How acceptable do you find the policy?; How much are you in favour of the new policy being introduced?; Do you support or oppose the new policy?”. Each was rated on a seven-point scale, labelled at either end: 1 = Strongly oppose; 7 = Strongly support.

*Secondary outcome:* The perceived effectiveness of the policy was measured with two items (*α* = 0.90) (Reynolds et al., 2018a): ”The new policy will reduce [*behaviour*]”; and, “The new policy will help solve England's problem with [*behaviour*]”. Each was rated on a seven-point scale labelled at either end: 1 = Strongly disagree, to 7 = Strongly agree.

*Demographic measures*: The research agency provided participant demographic data including age, gender, socio-economic status, education, and region. Educational achievement was recoded into three categories: low education (no education, GCSEs or similar); medium education (A-levels, non-degree teaching qualifications, or similar); and, high education (degree awards or higher). Socio-economic status was also recoded into three categories: low (DE), medium (C1C2), and high (AB). Indices of Multiple Deprivation (IMD) (McGuinness, 2015) were calculated based on the participant's constituency and were then recoded into quintiles: 1 = most deprived, 5 = least deprived.

*Behaviour and BMI:* Weekly alcohol consumption in units was measured with two items (*α* = 0.91) on a six-point scale (1 = under 1, 6 = more than 50). Daily tobacco use (*α* = 0.96) and daily vaping (*α* = 0.95) were measured with two items on a five-point scale (1 = 4 or fewer per day, to 5 = 31 + per day). High-calorie snacking was assessed with three items (*α* = 0.97) on an eight-point scale (1 = less than once a week, 8 = more than 4 a day) (Lally et al., 2011; Luszczynska et al., 2016). For an exploratory analysis, we categorised the alcohol, tobacco, and snacking variables into three groups: low, moderate, and heavy use. For alcohol, low use was non-drinkers, moderate use was 1–14 units per week (i.e. within the current UK safe drinking guidelines), and heavy use was more than 14 units per week. For tobacco, low use was non/former smokers, moderate use was non-daily smokers, and heavy use was daily smokers. For snacking we divided the variable into tertiles. BMI was calculated from participants' self-reported height and weight. See the supplement for the full questionnaire.

### 2.5 Analyses

The main analyses used full factorial ANOVAs to test the main effects and interactions between policy, behaviour, and evidence communication on acceptability and perceived effectiveness. Bonferroni adjusted pairwise comparisons were used to indicate if acceptability and perceived effectiveness significantly differed across the levels of each factor.

The predictors of acceptability were analysed in seven regression models (three behaviours and four policies). The ordinal variables that assessed frequency of consumption were not used in the regression analyses as few people reported smoking or vaping. These ordinal variables were replaced by dichotomous variables indicating whether or not participants currently smoked or vaped. A Bonferroni adjustment was applied to the seven regression models, *α* = 0.05/7 = 0.007. The model diagnostics were all acceptable.

Potential confounding variables (SES, gender, and age) were judged to be matched across groups using a percentage method to assess chance imbalances following the randomisation (Moher et al., 2010). The largest imbalance was 3 percentage points, 53% of the control group were female compared to 50% of those in the asserted evidence group.

To aid interpretation of the results, data in Table 1 were dichotomised (1–4 = 0, 4.01–7 = 1) to indicate the proportions of participants who found the policy acceptable (i.e. those rating above the scale midpoint). Outliers (±3SDs from the mean) on continuous variables were removed. There were no outliers in the primary or secondary outcome variables and therefore the planned main analyses were unaffected by outliers. 285 outliers from 7058 cases were removed from the high-calorie snacking variable (4%) and 269 were removed from the weekly alcohol consumption variable (5%).

**Table 1:** Acceptability (% (95% confidence intervals) [*n*]) for each policy by targeted behaviour, for participants not receiving any evidence of policy effectiveness.

	Alcohol consumption	Tobacco use	Snack consumption	Overall
Availability	44% (37%, 51%) [217]	62% (55%, 69%) [195]	33% (26%, 40%) [156]	47% (43%, 51%) [568]
Size	52% (45%, 59%) [188]	68% (62%, 74%) [209]	57% (50%, 64%) [220]	59% (55%, 63%) [617]
Labelling	76% (70%, 82%) [206]	89% (85%, 93%) [190]	71% (65%, 77%) [207]	78% (75%, 81%) [603]
Tax	48% (41%, 55%) [224]	74% (67%, 81%) [172]	50% (43%, 57%) [184]	57% (53%, 61%) [580]
Overall	55% (52%, 58%) [833]	73% (70%, 76%) [766]	54% (50%, 58%) [768]	60% (58%, 62%) [2368]

## 3 Results

### 3.1 Public acceptability

#### 3.1.1 Policy

A majority of participants found nudges and taxes to be acceptable overall (Table 1). These data are presented for the control group only *i.e.* for those who did not receive any evidence of effectiveness for the different interventions. This is to provide baseline data, from participants not influenced by evidence of policy effectiveness.

Policies that limited the availability of a product were the least acceptable, and the use of graphic labels on products were the most acceptable.

**Fig. 1:**
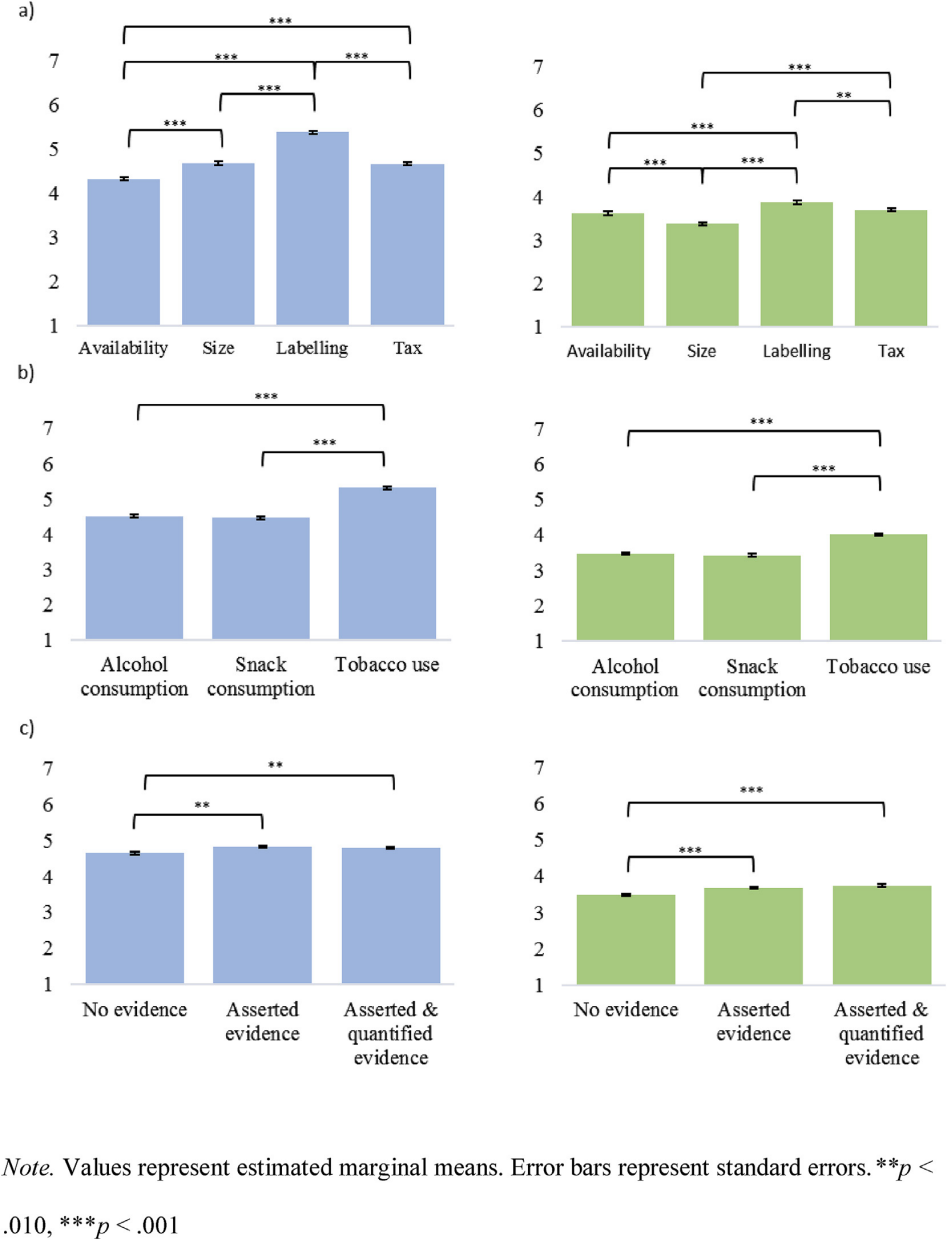
Acceptability (blue, left) and perceived effectiveness (green, right) by (a) Policy (b) Behaviour and (c) Communication of evidence. *Note.* Values represent estimated marginal means. Error bars represent standard errors.**p < .010, ***p < .001. (For interpretation of the references to colour in this figure legend, the reader is referred to the Web version of this article.)

Acceptability varied significantly across the four policy types, *F*(3, 7944) = 124.57, *p* < .001, *partial η*^2^ = 0.045. Fig. 1 shows the results of follow-up Bonferroni pairwise comparisons (see Table 2 for descriptive statistics).

**Table 2:** The acceptability (mean (SD) [*n*]) of each policy by behaviour and evidence communication group.

	Control			Asserted evidence			Asserted and quantified evidence		
	Alcohol	Tobacco	Food	Alcohol	Tobacco	Food	Alcohol	Tobacco	Food
Availability	4.15 (1.83) [217]	4.84 (2.03) [195]	3.57 (1.71) [156]	4.28 (1.83) [222]	5.07 (1.80) [193]	3.74 (1.85) [181]	4.23 (1.89) [194]	5.12 (1.94) [202]	3.80 (1.89) [215]
Size	4.16 (1.87) [188]	4.85 (1.72) [209]	4.43 (1.82) [220]	4.59 (1.84) [174]	5.15 (1.60) [191]	4.82 (1.80) [185]	4.41 (1.99) [231]	4.93 (1.85) [202]	4.83 (1.76) [197]
Labelling	5.08 (1.39) [206]	5.92 (1.27) [190]	5.05 (1.85) [207]	5.14 (1.56) [215]	5.77 (1.45) [168]	5.13 (1.64) [183]	5.23 (1.54) [180]	5.89 (1.34) [200]	5.33 (1.59) [193]
Tax	4.20 (1.79) [224]	5.38 (1.94) [172]	4.14 (1.76) [184]	4.50 (1.79) [196]	5.57 (1.72) [205]	4.32 (1.98) [204]	4.25 (1.88) [200]	5.46 (1.75) [178]	4.32 (1.91) [183]

*Note.* 7 point scale (1 = strongly oppose; 7 = strongly support).

#### 3.1.2 Behaviour

Policies that targeted tobacco use were the most acceptable. Policies that targeted alcohol use and high-calorie snack food consumption were less acceptable, and comparably acceptable.

Acceptability varied significantly and across the three behaviours, *F*(2, 7944) = 201.93, *p* < .001, *partial η*^2^ = 0.048 (see Fig. 1 and Table 2).

#### 3.1.3 Evidence communication

Relative to the control group, asserting evidence of effectiveness increased acceptability; adding a quantification to this assertion did not significantly increase this further. There was a significant main effect of communicating evidence of policy effectiveness on acceptability, *F*(2, 7944) = 9.24, *p* < .001, *partial η*^2^ = 0.002 (see Fig. 1 and Table 2). In comparison to the effect of policy and behaviour on support, the effect size (*partial η*^2^) of communicating evidence was small.

#### 3.1.4 Policy x behaviour x evidence communication interactions

There was a significant two-way interaction between Policy and Behaviour on acceptability, *F*(6, 7944) = 12.97, *p* < .001, *partial η*^2^ = 0.010. Exploration of this interaction using plots (see Fig. S1 in the supplement) suggested that the acceptability of Size policies did not follow the same pattern as the other policies across the three behaviours. Despite being the second most acceptable policy for Alcohol and Food, reducing the size of products was the least acceptable policy for Tobacco.

There were no significant two-way interactions between behaviour and evidence communication on acceptability, *F*(4, 7944) = 0.77, *p* = .544, *partial η*^2^ = 0.000, or between evidence communication and policy type on acceptability, *F*(6, 7944) = 1.40, *p* = .213, *partial η*^2^ = 0.001. The three-way interaction between evidence communication, behaviour and policy type on acceptability was not significant, *F*(12, 7944) = 0.26, *p* = .995, *partial η*^2^ = 0.000.

### 3.2 Perceived effectiveness

Beliefs about policy effectiveness varied significantly across the policies, *F*(3, 7944) = 36.54, *p* < .001, *partial η*^2^ = 0.014, and across the three behaviours, *F*(2, 7944) = 121.18, *p* < .001, *partial η*^2^ = 0.030. There was also a significant main effect of communicating evidence of policy effectiveness on beliefs about the effectiveness of the policy, *F*(2, 7944) = 21.76, *p* < .001, *partial η*^2^ = 0.005. Fig. 1 shows the results of follow-up Bonferroni pairwise comparisons (see also Table 3). Similar to the main effects on acceptability, the effects of policy and behaviour on perceived effectiveness were larger than the effect of communicating evidence on perceived effectiveness.

**Table 3:** The perceived effectiveness (mean (SD) [*n*]) of each policy by behaviour and evidence communication group.

	Control			Asserted evidence			Asserted and quantified evidence		
	Alcohol	Tobacco	Food	Alcohol	Tobacco	Food	Alcohol	Tobacco	Food
Availability	3.43 (1.63) [217]	3.99 (1.73) [195]	3.04 (1.48) [156]	3.58 (1.65) [222]	4.13 (1.67) [193]	3.14 (1.53) [181]	3.51 (1.58) [194]	4.29 (1.50) [202]	3.34 (1.58) [215]
Size	3.15 (1.63) [188]	3.20 (1.58) [209]	3.06 (1.47) [220]	3.50 (1.57) [174]	3.51 (1.56) [191]	3.36 (1.44) [185]	3.47 (1.65) [231]	3.64 (1.51) [202]	3.59 (1.51) [197]
Labelling	3.45 (1.44) [206]	4.03 (1.49) [190]	3.77 (1.66) [207]	3.67 (1.54) [215]	4.45 (1.38) [168]	3.59 (1.37) [183]	3.81 (1.41) [180]	4.18 (1.42) [200]	4.07 (1.51) [193]
Tax	3.28 (1.6) [224]	4.24 (1.59) [172]	3.26 (1.52) [184]	3.53 (1.60) [196]	4.38 (1.68) [205]	3.43 (1.69) [204]	3.41 (1.45) [200]	4.40 (1.56) [178]	3.47 (1.59) [183]

*Note.* 7 point scale (1 = strongly oppose; 7 = strongly support).

There was a significant two-way interaction between behaviour and policy type on beliefs about the effectiveness of the policy, *F*(6, 7944) = 15.69, *p* < .001, *partial η*^2^ = 0.012. Exploration of this interaction using plots (see Fig. S2 in the supplement) suggested that the perceived effectiveness of Size policies did not follow the same pattern as the other policies across the three behaviours. Unlike the other three policies, the perceived effectiveness of reducing the size of products showed little variation across behaviours; this policy was consistently believed to be ineffective.

There were no significant two-way interactions between behaviour and evidence communication on beliefs about the effectiveness of the policy, *F*(4, 7944) = 1.53, *p* = .190, *partial η*^2^ = 0.001, or between evidence communication and policy type on beliefs about the effectiveness of the policy, *F*(6, 7944) = 1.21, *p* = .299, *partial η*^2^ = 0.001. The three-way interaction between evidence communication, behaviour and policy type on beliefs about the effectiveness of the policy was not significant, *F*(12, 7944) = 1.24, *p* = .249, *partial η*^2^ = 0.002.

### 3.3 Predictors of acceptability

The perceived effectiveness of a policy was the strongest predictor of its acceptability across all seven regression models (See Table 4, Table 5 for full models). The most reliable demographic predictor of policy support was gender, with women judging policies to be more acceptable in six of the seven models. Indices of socio-economic position – education, IMD, and social grade – were mostly unrelated to acceptability. There were several exceptions to this, participants with high education judged policies to change the size of products more acceptable than did those with low education, *B* = 0.25, *p* = .006, and participants living in the most deprived areas had less support for policies targeting unhealthy snack foods (see Table 4). Other predictors of acceptability for policies targeting different behaviours included engagement in the targeted behaviour. People who reported that they currently smoked (*B* = −1.08, *p* < .001) or vaped (*B* = −0.48, *p* < .001) judged policies that aimed to reduce tobacco use less acceptable. People who ate more high-calorie snacks were less accepting of policies to reduce high-calorie snacking, *B* = −0.02, *p* = .006, and people who drank more alcohol were less accepting of policies to reduce alcohol use, *B* = −0.02, *p* < .001.

**Table 4:** Regression models predicting acceptability for the three behaviours.

	Alcohol (*n* = 1643)		Tobacco (*n* = 1552)		Food (*n* = 1576)	
	*B* (SE)	*p*	*B* (SE)	*p*	*B* (SE)	*p*
(Intercept)	0.70 (0.26)	.007	2.39 (0.27)	< .001	0.24 (0.28)	.394
Policy group (tax)	0.23 (0.08)	.006	0.39 (0.09)	< .001	0.41 (0.09)	< .001
Policy group (size)	0.27 (0.08)	.001	0.57 (0.09)	< .001	0.93 (0.09)	< .001
Policy group (labelling)	0.88 (0.08)	< .001	0.91 (0.09)	< .001	1.03 (0.09)	< .001
Evidence group (Asserting)	0.01 (0.07)	.884	-0.01 (0.08)	.900	0.03 (0.08)	.717
Evidence group (Assert. & quantifying)	-0.02 (0.07)	.759	-0.02 (0.07)	.787	0.00 (0.08)	.969
Age (years)	0.01 (0.00)	.004	-0.01 (0)	.003	0.00 (0.00)	.263
Gender (F)	0.21 (0.06)	< .001	0.18 (0.06)	.004	0.21 (0.06)	.001
Education (high)	0.07 (0.08)	.393	0.00 (0.08)	.954	0.13 (0.09)	.139
Education (medium)	0.07 (0.07)	.371	-0.18 (0.08)	.015	0.07 (0.08)	.350
Social grade (AB)	-0.08 (0.09)	.370	0.16 (0.09)	.082	0.17 (0.10)	.084
Social grade (C1C2)	-0.10 (0.08)	.232	0.05 (0.09)	.587	0.06 (0.09)	.522
IMD Q2	0.08 (0.10)	.396	0.10 (0.10)	.324	0.35 (0.10)	.001
IMD Q3	0.12 (0.10)	.204	0.19 (0.10)	.058	0.23 (0.10)	.024
IMD Q4	0.23 (0.10)	.014	-0.02 (0.10)	.854	0.32 (0.10)	.002
IMD Q5	0.09 (0.10)	.370	0.09 (0.10)	.384	0.33 (0.11)	.002
BMI (kg/m^2^)	0.01 (0.01)	.202	0.01 (0.01)	.227	0.01 (0.01)	.159
Unhealthy snacks per week	-0.01 (0.01)	.031	-0.02 (0.01)	< .001	-0.02 (0.01)	.007
Unit of alcohol per week	-0.02 (0.00)	< .001	0.00 (0.00)	.307	-0.01 (0.00)	.201
Current smoker (yes)	-0.19 (0.09)	.036	-1.08 (0.10)	< .001	-0.01 (0.10)	.959
Current vaper (yes)	-0.08 (0.11)	.482	-0.48 (0.13)	< .001	-0.23 (0.13)	.073
Perceived effectiveness	0.81 (0.02)	< .001	0.66 (0.02)	< .001	0.81 (0.02)	< .001
Adjusted *R*^2^	.56		.52		.55	

*Note*. After a Bonferroni adjustment, significant effects are those in which *p* < .007. Policy group: Availability is the reference category. Evidence group: control (no evidence) is the reference category. Gender: men is the reference category. Social grade: DE is the reference category. Education: Low education is the reference category. Current smoker/vaper: non-smokers/vapers are the reference categories. IMD Q = Indices of Multiple Deprivation Quintiles. IMD Q1 (the most deprived) is the reference category. All other variables are continuous.

**Table 5:** Regression models predicting acceptability for the four policies.

	Availability (*n* = 1653)		Size (*n* = 1683)		Labelling (*n* = 1605)		Tax (*n* = 1615)	
	*B* (SE)	*p*	*B* (SE)	*p*	*B* (SE)	*p*	*B* (SE)	*p*
(Intercept)	0.65 (0.27)	.016	0.35 (0.26)	.178	1.92 (0.24)	< .001	0.77 (0.25)	.002
Behaviour group (tobacco)	0.27 (0.08)	.001	0.52 (0.08)	< .001	0.39 (0.07)	< .001	0.40 (0.08)	< .001
Behaviour group (food)	-0.22 (0.08)	.006	0.33 (0.08)	< .001	-0.05 (0.07)	.450	-0.06 (0.08)	.449
Evidence group (Asserting)	0.07 (0.08)	.376	0.16 (0.08)	.053	-0.11 (0.07)	.144	0.08 (0.08)	.273
Evidence group (Assert. & quantifying)	0.03 (0.08)	.699	-0.06 (0.08)	.416	-0.05 (0.07)	.533	-0.03 (0.08)	.690
Age (years)	0.00 (0.00)	.129	0.00 (0.00)	.639	0.00 (0.00)	.026	0.00 (0.00)	.469
Gender (F)	0.23 (0.07)	< .001	0.46 (0.06)	< .001	0.29 (0.06)	< .001	0.16 (0.06)	.014
Education (high)	-0.11 (0.09)	.225	0.25 (0.09)	.006	0.02 (0.08)	.805	0.12 (0.09)	.163
Education (medium)	-0.08 (0.08)	.332	0.05 (0.08)	.503	-0.01 (0.07)	.842	0.04 (0.08)	.588
Social grade (AB)	0.13 (0.10)	.173	-0.05 (0.10)	.600	0.19 (0.09)	.033	0.21 (0.10)	.031
Social grade (C1C2)	-0.01 (0.08)	.930	-0.03 (0.09)	.715	0.14 (0.08)	.081	0.04 (0.08)	.629
IMD Q2	0.16 (0.11)	.122	0.21 (0.11)	.046	0.21 (0.10)	.033	-0.07 (0.10)	.488
IMD Q3	0.21 (0.10)	.038	0.24 (0.10)	.020	-0.01 (0.10)	.936	-0.01 (0.10)	.949
IMD Q4	0.14 (0.11)	.186	0.15 (0.10)	.158	0.20 (0.10)	.050	-0.04 (0.10)	.732
IMD Q5	0.15 (0.10)	.161	0.25 (0.11)	.017	0.08 (0.10)	.401	0.06 (0.11)	.561
BMI (kg/m^2^)	0.00 (0.01)	.823	0.01 (0.01)	.036	0.01 (0.01)	.214	0.01 (0.01)	.061
Perceived effectiveness	0.82 (0.02)	< .001	0.80 (0.02)	.001	0.61 (0.02)	< .001	0.83 (0.02)	< .001
Adjusted *R*^2^	.54		.49		.41		.56	

*Note*. After a Bonferroni adjustment, significant effects are those in which *p* < .007. Behaviour group: Alcohol is the reference category. Evidence group: control (no evidence) is the reference category. Gender: men is the reference category. Social grade: DE is the reference category. Education: Low education is the reference category. Current smoker/vaper: non-smokers/vapers are the reference categories. IMD Q = Indices of Multiple Deprivation Quintiles. IMD Q1 (the most deprived) is the reference category. All other variables are continuous.

**Fig. 2:**
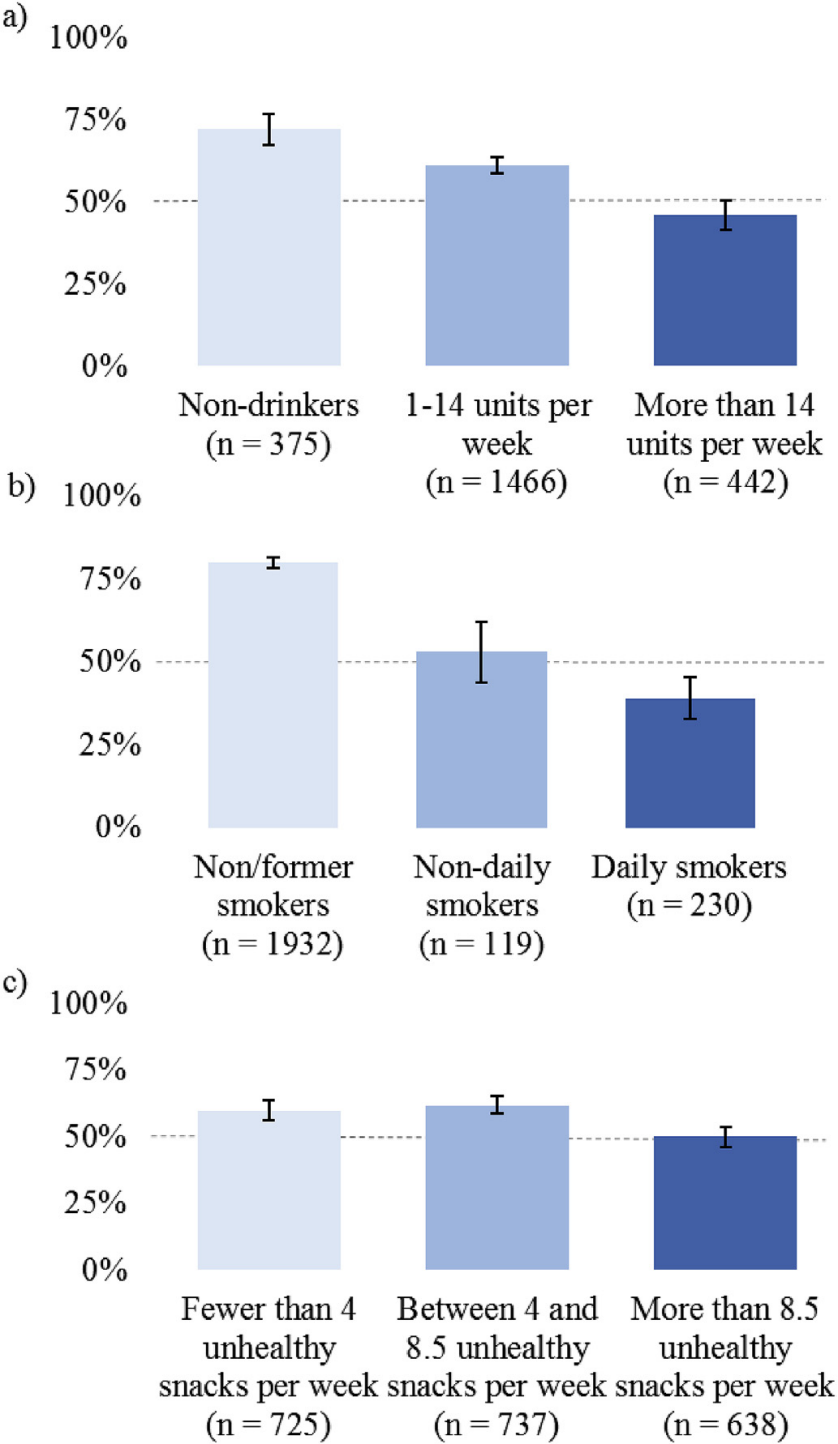
Proportion (%) supporting policies targeting (a) alcohol (b) tobacco and (c) unhealthy snacks and level of consumption for each product. *Note.* Error bars represent 95% confidence intervals.

In an exploratory analysis, we divided the sample into three categories (low/no use, moderate use, or frequent use of the three different products (see Fig. 2) to explore the degree to which acceptability decreases among people with more frequent consumption of the target product. A minority of heavy alcohol users found policies targeting alcohol use acceptable, (46%, 95% CI [41%, 51%]), a minority of daily tobacco users found policies targeting tobacco use acceptable (39%, 95% CI [33%, 45%]), and around half of frequent snackers found policies targeting unhealthy snacks acceptable (50%, 95% CI [46%, 54%]).

## 4 Discussion

The current study aimed to estimate the acceptability of four policies for changing three unhealthy behaviours, and to assess whether communicating evidence of policy effectiveness could increase the acceptability of these policies. The majority of participants found nudges acceptable, replicating results from many countries around the world (Petrescu et al., 2016; Reisch et al., 2017; Sunstein, Reisch and Kaiser, 2018a; Sunstein, Reisch and Rauber, 2018b). Levels of acceptability differed, however, across policies and behaviours. Adding graphic warning labels to products was the most acceptable policy, followed by taxation and changing product size, which received similar levels of support. Limiting the availability of products by banning them in corner shops was the least acceptable policy. The most acceptable behaviour targeted by the policies was tobacco use. Acceptability of policies to tackle alcohol use and high-calorie snacking were similar, and much lower than that for tobacco use. Asserting evidence that the policies were effective increased their acceptability; quantifying the effectiveness did not add to this.

Acceptability differed across the four policies and, as predicted, graphic warning labels were significantly more acceptable than the other three. The availability policy – banning the product in local shops - was the least acceptable. It should be noted, however, that this is a highly restrictive variant of interventions targeting availability. This replicates previous findings that highlight that acceptability is lower among more intrusive policy options (Diepeveen et al., 2013; Hagmann et al., 2018; Nuffield Council on Bioethics, 2007; Petrescu et al., 2016). Providing information may be perceived as not impinging on choice in contrast to increasing the price, limiting the size of products, or reducing their availability. Maintaining a perception of choice is core to perceptions of fairness (Mattila and Cranage, 2005), which, together with perceived effectiveness, are the two main predictors of acceptability (Bos et al., 2015; Reynolds et al., 2018a; Schuitema et al., 2011).

Acceptability also differed across behaviours. Participants judged the same policies as more acceptable when applied to tobacco use, whereas policies targeting alcohol and snack consumption were deemed similarly acceptable. This high support for tobacco use interventions accords with a recent review on the acceptability of health interventions (Diepeveen et al., 2013). Such high support likely reflects the fact that most people in high income countries – from where most of this evidence derives - are not current smokers (Ng et al., 2014). As shown in the current study, those not engaging in a behaviour support policies targeting that behaviour more than do those who engage in the behaviour. There was no difference in acceptability for policies applied to either alcohol or snack consumption. Policies that targeted these two behaviours were judged to be acceptable by a slim majority of the sample, around 54–55%. These estimates are similar to previous studies investigating the acceptability of policies to tackle alcohol use (Li et al., 2017; Pechey et al., 2014), but there are limited data on the acceptability of policies to reduce unhealthy snack food consumption (Reynolds et al., 2018a), and therefore the current study provides valuable new information. There was a two-way interaction between Policy and Behaviour on acceptability and perceived effectiveness. In both cases, it appeared that the Size policy was less variable across the three behaviours. Any other policy applied to Tobacco received more support than if the same policy was applied to Alcohol or Food. However, with the Size policy, acceptability and perceived effectiveness either did not increase as much as would be expected, or not at all.

In keeping with other studies (Bos et al., 2015; Storvoll et al., 2015), perceived effectiveness was the strongest predictor of acceptability in the current study. As found in previous experimental studies, communicating evidence that policies were effective at changing behaviour increased their acceptability by only three to five percentage points (Reynolds et al., 2018a; Reynolds et al., Under review). Reconciling this strong correlation with a weak experimental effect can be done when considering two factors. First, a majority of participants reported that the policy would not be effective despite reading evidence that it would be effective. There is a large body of evidence that demonstrates that a proportion of people will not update their beliefs when confronted with evidence and this may be in part explained by motivated reasoning or the confirmation bias (Chan et al., 2017; Druckman and McGrath, 2019; Gilead et al., 2018; Nickerson, 1998; Nyhan and Reifler, 2015; Reynolds et al., 2018a). These biases could explain why the experimental effect is weak. Second, a reverse causation explanation may explain part of why the correlation is strong, in other words, acceptability predicts effectiveness (i.e. a judgement to support a policy leads people to believe the policy is effective, while a judgement to not support a policy leads people to believe the policy is ineffective). Although there is no direct evidence for this interpretation, it is consistent with people attempting to reduce cognitive dissonance that may arise from judging an ineffective policy as acceptable (Shultz and Lepper, 1996). The small effect of communicating evidence on acceptability, while important, highlights that merely communicating evidence of effectiveness would be insufficient to generate strong public support for a specific policy. It does however provide evidence for the effectiveness of one component of a broad set of advocacy activities. This is highly pertinent in the context of industry opposition to such interventions and the reluctance of many politicians to support measures which have the potential to improve population health, but which they fear may be unpopular with the wider public (Donaldson, 2015; Roache and Gostin, 2017, 2018). As the relationship between public support and policy implementation is complex and not readily amenable to investigation using scientific methods, the contribution which communicating evidence of policy effectiveness makes to policy outcomes is not straightforward. Harnessing popular support has been identified as a key factor in developing and implementing policies (Cairney, 2009; Cullerton et al., 2016; Cullerton et al., 2018). However, the precise level of support needed to influence policy makers to deliver a policy is unknown and likely to vary by policy, time, and jurisdiction.

The current study builds on the existing literature on evidence communication by comparing the two forms in which evidence of effectiveness is often communicated, namely by asserting effectiveness (e.g., “Research shows that the introduction of this policy will reduce the number of people who smoke”) and quantifying effectiveness (e.g., “Research shows that the introduction of this policy will reduce the number of people who smoke by 10%”). Quantifying the effectiveness did not clearly confer any additional benefit over simply asserting that the policy was effective. There was no difference in perceptions of policy effectiveness or support for a policy between these two groups. There are several possible explanations for these findings. First, people may be insensitive to numbers indicating magnitude of an effect. Evidence against this explanation comes from a discrete choice experiment which found acceptability of interventions was sensitive to numbers denoting magnitude of effects (Promberger et al., 2012). Second, people may be sensitive to numbers but any impact will depend upon people's pre-existing estimates of effectiveness. For example, some participants may hold a belief that the policy would change behaviour by 10%, others by 20% and yet others by 0%. This could explain the apparent null effect on aggregate ratings for perceived effectiveness of quantification. Assessing participants' pre-existing estimates could enable this to be explored in future studies.

The regression models suggest that people who engage in a behaviour reliably oppose policies to reduce that behaviour. Tobacco users and vapers were less accepting of policies targeting tobacco use, and acceptability of policies to targeting both alcohol use and snack foods decreased as weekly use of the respective product increased. Fig. 2 illustrates this further, showing that support for a policy decreases as consumption increases of the product at which the policy is aimed. This pattern is most marked for tobacco and alcohol. This pattern of results echoes those from previous studies showing that support for interventions is lower amongst those whose behaviour is targeted, including those who consume more alcohol and consume sugar sweetened beverages (Hagmann et al., 2018; Pechey et al., 2014; Sunstein, Reisch and Kaiser, 2018a). The decline in support from those most affected by a policy touches on a broader question about the legitimacy of policies that are favoured by majorities but which appear to impinge upon the values and behaviour of minorities (Waldron, 1990).

These regression analyses also show that women were more likely to support the policies than were men, consistent with previous studies (Diepeveen et al., 2013; Reisch et al., 2017). Diepeveen et al. (2013) speculated that it might reflect the observed higher concern about health expressed by women, compared with men (Wardle et al., 2004) or a higher value placed on reducing the need for caring arising from the higher prevalence of women as carers. These and other hypotheses await testing.

Socio-economic status, as assessed by educational achievement, social grade, and the Index of Multiple Deprivation had little association with acceptability. One exception to this was the acceptability of policies to change the size of products: those with high levels of education supported these policies more so than those with low levels of education. A second exception to this was stronger support for policies to reduce unhealthy snack consumption among those living in less deprived areas (i.e. better health outcomes, lower crime, wealthier). It is not clear why these two results were significant, however the main pattern of results in the current study – an absence of an association between socio-economic status and acceptability of policies to change behaviour to improve health – is reported in other studies (Hagmann et al., 2018; Pechey et al., 2014; Petrescu et al., 2016). In a recent study assessing public attitudes towards health, wealth and political inequalities, again, no social patterning was evident in these attitudes (Howarth et al., 2019). Health equity was valued highly regardless of socio-economic position, which may contribute to the absence of a social patterning in the acceptability of interventions to improve health observed in the current study.

A strength of the current study is the full between-participants design. This ensures that each estimate of acceptability is not influenced by prior exposure to questions about the acceptability of other policies, and participants are also less likely to be affected by fatigue effects. A limitation of this design is that, despite the large overall sample size, the sample size per cell is smaller than in some similar studies and therefore each estimate has larger confidence intervals (Hagmann et al., 2018; Reisch et al., 2017). A further limitation is that the policies were only described in a short text statement. These estimates - particularly for the graphic warning labels - may be less accurate than if the actual warning label was shown to participants. This was not done to ensure that the groups were as comparable as possible.

The current study estimated acceptability in England only and although some countries have similar attitudes toward nudges - such as France or the USA - others do differ (Petrescu et al., 2016; Reisch et al., 2017). Future studies could continue to assess acceptability and factors affecting its variation across countries, extending existing evidence in high-income countries to include those in middle and low-income ones. Alcohol consumption, tobacco use, and high-calorie snacking are three important behaviours that affect health outcomes (Lim et al., 2012; Steel et al., 2018; Whiteford et al., 2013). Given that acceptability of policies differed across the three sets of behaviour that were the focus of the current study, acceptability of other behaviours that affect population and planetary health – such as consumption of red and processed meat – cannot be assumed. Further study of such behaviours are therefore warranted.

In conclusion, the majority of the study sample perceived nudging and taxing to be acceptable for changing unhealthy behaviours to improve population health. There was, however, considerable variation by policy and behaviour. All four policies targeting tobacco, including banning tobacco sales in corner shops, received majority support. Three policies targeting snacking and two targeting alcohol consumption received majority support. This highlights that there is already public will for stronger government action to improve population health. Furthermore, communicating evidence of their effectiveness increased support for policies that can reduce overconsumption of alcohol, cigarettes, and unhealthy foods. This suggests that highlighting effectiveness could contribute to mobilising public demand for policies. While uncertainty remains about the strength of public support needed, this may help overcome political inertia and enable action on behaviours that damage population and planetary health.
