# Supplementary material for: Public acceptability of nudging and taxing to reduce consumption of alcohol, tobacco, and food: A population-based survey experiment
Source: Soc Sci Med. 2019 Sep;236:112395. doi: 10.1016/j.socscimed.2019.112395 (PMC6695289; doi:10.1016/j.socscimed.2019.112395)

**Public acceptability of nudging and taxing to reduce consumption of alcohol, tobacco, and food: A population-based survey experiment**

Table S1. Weighted demographic characteristics of the sample by policy (%)

|  | Availability  (*n* = 1775) | Tax  (*n* = 1745) | Size  (*n* = 1797) | Labelling  (*n* = 1741) |
| --- | --- | --- | --- | --- |
| Gender |  |  |  |  |
| Male | 47 | 48 | 50 | 49 |
| Female | 53 | 52 | 50 | 51 |
| Age |  |  |  |  |
| 18-24 | 10 | 11 | 12 | 12 |
| 25-34 | 14 | 15 | 14 | 14 |
| 35-44 | 17 | 18 | 18 | 17 |
| 45-54 | 18 | 19 | 16 | 19 |
| 55+ | 40 | 37 | 40 | 38 |
| Socioeconomic status |  |  |  |  |
| AB | 22 | 21 | 21 | 20 |
| C1C2 | 51 | 51 | 50 | 51 |
| DE | 27 | 28 | 29 | 29 |

Table S2. Weighted demographic characteristics of the sample by behaviour (%)

|  | Alcohol  (*n* = 2444) | Tobacco  (*n* = 2305) | Food  (*n* = 2309) |
| --- | --- | --- | --- |
| Gender |  |  |  |
| Male | 48 | 49 | 48 |
| Female | 52 | 51 | 52 |
| Age |  |  |  |
| 18-24 | 11 | 11 | 11 |
| 25-34 | 15 | 14 | 14 |
| 35-44 | 17 | 18 | 17 |
| 45-54 | 18 | 18 | 19 |
| 55+ | 39 | 38 | 40 |
| Socioeconomic status |  |  |  |
| AB | 21 | 21 | 21 |
| C1C2 | 51 | 49 | 52 |
| DE | 28 | 29 | 27 |

|  |  |
| --- | --- |

Table S3. Weighted demographic characteristics of the sample by evidence communication (%)

|  | Control (no message)  (*n* = 2368) | Asserted evidence  (*n* = 2317) | Asserted and quantified evidence  (*n* = 2373) | Total sample  (*N* = 7058) |
| --- | --- | --- | --- | --- |
| Gender |  |  |  |  |
| Male | 47 | 50 | 49 | 49 |
| Female | 53 | 50 | 51 | 51 |
| Age |  |  |  |  |
| 18-24 | 12 | 12 | 10 | 11 |
| 25-34 | 14 | 14 | 14 | 14 |
| 35-44 | 16 | 19 | 17 | 17 |
| 45-54 | 18 | 18 | 19 | 18 |
| 55+ | 40 | 37 | 40 | 39 |
| Socioeconomic status |  |  |  |  |
| AB | 21 | 22 | 21 | 21 |
| C1C2 | 51 | 50 | 52 | 51 |
| DE | 28 | 28 | 27 | 28 |

**Figure S1.** Plots exploring the two-way interaction between Policy and Behaviour on public acceptability


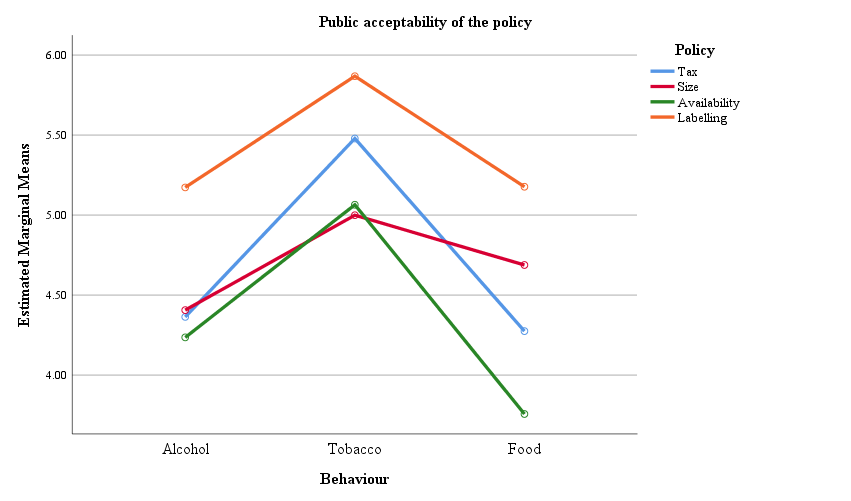


**Figure S2.** Plots exploring the two-way interaction between Policy and Behaviour on perceived effectiveness


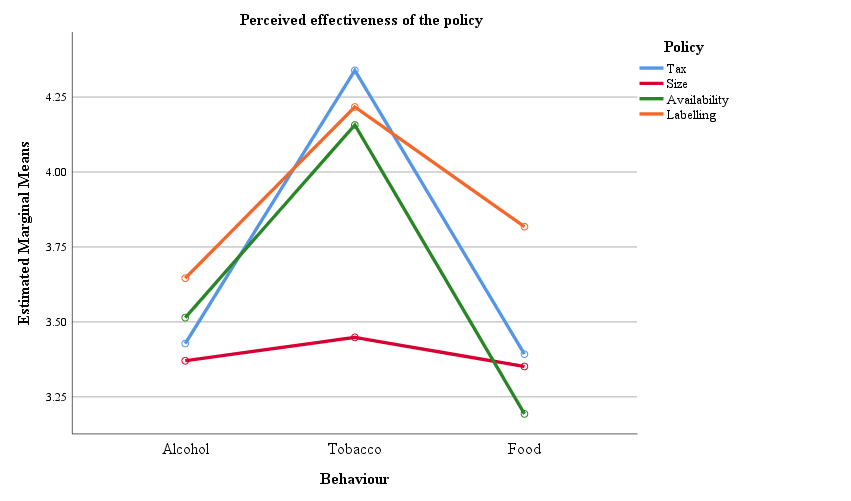

Supplement: Multimedia component 1 [file mmc1.docx]
