# Supplementary material for: Public acceptability of nudging and taxing to reduce consumption of alcohol, tobacco, and food: A population-based survey experiment
Source: Soc Sci Med. 2019 Sep;236:112395. doi: 10.1016/j.socscimed.2019.112395 (PMC6695289; doi:10.1016/j.socscimed.2019.112395)
Supplement: Multimedia component 2 [file mmc2.docx]

**Public acceptability of nudging and taxing to reduce consumption of alcohol, tobacco, and food: A population-based survey experiment**

The full factorial design used in this study has 36 conditions (see Table 1). Participants will be allocated to one of the 36 groups using stratified random sampling. The 36 numbered policy statements correspond to Table 1.

Table 1 - Full factorial design matrix detailing all 36 conditions

|  | Control | | | | Asserting | | | | Asserting + Quantifying | | | |
| --- | --- | --- | --- | --- | --- | --- | --- | --- | --- | --- | --- | --- |
|  | Tax | Size | Avail | Label | Tax | Size | Avail | Label | Tax | Size | Avail | Label |
| Alcohol | 1 | 4 | 7 | 10 | 2 | 5 | 8 | 11 | 3 | 6 | 9 | 12 |
| Tobacco | 13 | 16 | 19 | 22 | 14 | 17 | 20 | 23 | 15 | 18 | 21 | 24 |
| Food | 25 | 28 | 31 | 34 | 26 | 29 | 32 | 35 | 27 | 30 | 33 | 36 |

Alcohol, Excess drinking, Tax

1. Control: The government is considering a new policy to increase the price of alcohol to help people drink less.
2. Asserted: The government is considering a new policy to increase the price of alcohol to help people drink less. Research shows that the introduction of this new policy will reduce the number of people who drink in ways that harm their health.
3. Asserted + quantified: The government is considering a new policy to increase the price of alcohol to help people drink less. Research shows that the introduction of this new policy will reduce the number of people who drink in ways that harm their health by 10%.

Alcohol, Excess drinking, Size

1. Control: The government is considering a new policy to reduce the serving size of alcoholic drinks pubs and restaurants to help people drink less. The price will be reduced in line with the change in size.
2. Asserted: The government is considering a new policy to reduce the serving size of alcoholic drinks in pubs and restaurants to help people drink less. The price will be reduced in line with the change in size. Research shows that the introduction of this new policy will reduce the number of people who drink in ways that harm their health.
3. Asserted + quantified: The government is considering a new policy to reduce the serving size of alcoholic drinks in pubs and restaurants to help people drink less. The price will be reduced in line with the change in size. Research shows that the introduction of this new policy will reduce the number of people who drink in ways that harm their health by 10%.

Alcohol, Excess drinking, Availability

1. Control: The government is considering a new policy to ban the sale of alcohol in corner shops to help people drink less.
2. Asserted: The government is considering a new policy to ban the sale of alcohol in corner shops to help people drink less. Research shows that the introduction of this new policy will reduce the number of people who drink in ways that harm their health.
3. Asserted + quantified: The government is considering a new policy to ban the sale of alcohol in corner shops to help people drink less. Research shows that the introduction of this new policy will reduce the number of people who drink in ways that harm their health by 10%.

Alcohol, Excess drinking, Labelling

1. Control: The government is considering a new policy to add graphic warning labels to alcohol to help people drink less.
2. Asserted: The government is considering a new policy to add graphic warning labels to alcohol to help people drink less. Research shows that the introduction of this new policy will reduce the number of people who drink in ways that harm their health.
3. Asserted + quantified: The government is considering a new policy to add graphic warning labels to alcohol to help people drink less. Research shows that the introduction of this new policy will reduce the number of people who drink in ways that harm their health by 10%.

Tobacco, Smoking, Tax

1. Control: The government is considering a new policy to increase the price of cigarettes to help people stop smoking.
2. Asserted: The government is considering a new policy to increase the price of cigarettes to help people stop smoking. Research shows that the introduction of this new policy will reduce the number of people who smoke.
3. Asserted + quantified: The government is considering a new policy to increase the price of cigarettes to help people stop smoking. Research shows that the introduction of this new policy will reduce the number of people who smoke by 10%.

Tobacco, Smoking, Size

1. Control: The government is considering a new policy to reduce the number of cigarettes in a pack to help people stop smoking. The price will be reduced in line with the change in size.
2. Asserted: The government is considering a new policy to reduce the number of cigarettes in a pack to help people stop smoking. The price will be reduced in line with the change in size. Research shows that the introduction of this new policy will reduce the number of people who smoke.
3. Asserted + quantified: The government is considering a new policy to reduce the number of cigarettes in a pack to help people stop smoking. The price will be reduced in line with the change in size. Research shows that the introduction of this new policy will reduce the number of people who smoke by 10%.

Tobacco, Smoking, Availability

1. Control: The government is considering a new policy to ban the sale of cigarettes in corner shops to help people stop smoking.
2. Asserted: The government is considering a new policy to ban the sale of cigarettes in corner shops to help people stop smoking. Research shows that the introduction of this new policy will reduce the number of people who smoke.
3. Asserted + quantified: The government is considering a new policy to ban the sale of cigarettes in corner shops to help people stop smoking. Research shows that the introduction of this new policy will reduce the number of people who smoke by 10%.

Tobacco, Smoking, Labelling

1. Control: The government is considering a new policy to add graphic warning labels to cigarettes to help people stop smoking.
2. Asserted: The government is considering a new policy to add graphic warning labels to cigarettes to help people stop smoking. Research shows that the introduction of this new policy will reduce the number of people who smoke.
3. Asserted + quantified: The government is considering a new policy to add graphic warning labels to cigarettes to help people stop smoking. Research shows that the introduction of this new policy will reduce the number of people who smoke by 10%.

Food, Obesity, Tax

1. Control: The government is considering a new policy to increase the price of high calorie snacks (e.g. crisps and sweets) to tackle obesity.
2. Asserted: The government is considering a new policy to increase the price of high calorie snacks (e.g. crisps and sweets) to tackle obesity. Research shows that the introduction of this new policy will reduce the number of people who are overweight or obese.
3. Asserted + quantified: The government is considering a new policy to increase the price of high calorie snacks (e.g. crisps and sweets) to tackle obesity. Research shows that the introduction of this new policy will reduce the number of people who are overweight or obese by 10%.

Food, Obesity, Size

1. Control: The government is considering a new policy to reduce the size of packets of high calorie snacks (e.g. crisps and sweets) to tackle obesity. The price will be reduced in line with the change in size.
2. Asserted: The government is considering a new policy to reduce the size of packets of high calorie snacks (e.g. crisps and sweets) to tackle obesity. The price will be reduced in line with the change in size. Research shows that the introduction of this new policy will reduce the number of people who are overweight or obese.
3. Asserted + quantified: The government is considering a new policy to reduce the size of packets of high calorie snacks (e.g. crisps and sweets) to tackle obesity. The price will be reduced in line with the change in size. Research shows that the introduction of this new policy will reduce the number of people who are overweight or obese by 10%.

Food, Obesity, Availability

1. Control: The government is considering a new policy to ban the sale of high calorie snacks (e.g. crisps and sweets) in corner shops to tackle obesity.
2. Asserted: The government is considering a new policy to ban the sale of high calorie snacks (e.g. crisps and sweets) in corner shops to tackle obesity. Research shows that the introduction of this new policy will reduce the number of people who are overweight or obese.
3. Asserted + quantified: The government is considering a new policy to ban the sale of high calorie snacks (e.g. crisps and sweets) in corner shops to tackle obesity. Research shows that the introduction of this new policy will reduce the number of people who are overweight or obese by 10%.

Food, Obesity, Labelling

1. Control: The government is considering a new policy to add graphic warning labels to high calorie snacks (e.g. crisps and sweets) to tackle obesity.
2. Asserted: The government is considering a new policy to add graphic warning labels to high calorie snacks (e.g. crisps and sweets) to tackle obesity. Research shows that the introduction of this new policy will reduce the number of people who are overweight or obese.
3. Asserted + quantified: The government is considering a new policy to add graphic warning labels to high calorie snacks (e.g. crisps and sweets) to tackle obesity. Research shows that the introduction of this new policy will reduce the number of people who are overweight or obese by 10%.

**Appendix 2 – Survey questions**

**[One of the 36 different policy statements to be displayed here]**

[Q1] How acceptable do you find the new policy?

<1>Completely acceptable

<2>Acceptable

<3>A little acceptable

<4>Neither unacceptable nor acceptable

<5>A little unacceptable

<6>Unacceptable

<7>Completely unacceptable

[Q2] Do you support or oppose the new policy?

<1>Strongly support

<2>Support

<3>Support a little

<4>Neither oppose nor support

<5>Oppose a little

<6>Oppose

<7>Strongly oppose

[Q3] How much are you in favour of the new policy being introduced?

<1>Strongly in favour

<2>In favour

<3>A little in favour

<4>Neither against nor in favour

<5>A little against

<6>Against

<7>Strongly against

*Secondary Outcome Questions:*

**To what extent do you agree or disagree with the following statements?**

[Q4] The new policy will reduce [behaviour x1]

<1>Strongly agree

<2>Agree

<3>Somewhat agree

<4>Neither agree nor disagree

<5>Somewhat disagree

<6>Disagree

<7>Strongly disagree

[Q5] The new policy will help solve England’s problem with [behaviour x2]

<1>Strongly agree

<2>Agree

<3>Somewhat agree

<4>Neither agree nor disagree

<5>Somewhat disagree

<6>Disagree

<7>Strongly disagree

*‘Demographics’:*

[Q6] How tall are you?

<1>Dropdown box

<2>Don’t know

<3>Prefer not to say

[Q7] How much do you weigh? With a ‘DK’ and a ‘Prefer not to say’

<1>Dropdown box

<2>Don’t know

<3>Prefer not to say

[Q8] Which, if any, of the following statements BEST applies to you, when it comes to smoking?

<1>I smoke every day

<2>I smoke but I don’t smoke every day

<3>I used to smoke but I have given up now

<4>I have never smoked

<5>Prefer not to say

##If selected 1 or 2 in Q8

[Q8a] Approximately how many cigarettes did you smoke yesterday?

<1>4 or fewer

<2>5-10

<3>11-20

<4>21-30

<5>31+

<6>Don’t know

##If selected 1 or 2 in Q8

[Q8b] Approximately how many cigarettes do you typically smoke on an average day?

<1>4 or fewer

<2>5-10

<3>11-20

<4>21-30

<5>31+

<6>Don’t know

[Q9] Which, if any, of the following statements BEST applies to you, when it comes to vaping? By ‘vaping’ we mean inhaling vapour from electronic cigarettes and other similar devices (e.g. vaporisers etc.).

<1>I vape every day

<2>I vape but I don’t vape every day

<3>I used to vape but I have given up now

<4>I have never vaped

<5>Prefer not to say

##If selected 1 or 2 in Q9

[Q9a] Approximately how many times did you vape yesterday?

<1>4 or fewer

<2>5-10

<3>11-20

<4>21-30

<5>31+

<6>Don’t know

##If selected 1 or 2 in Q9

[Q9b] Approximately how many times do you typically vape on an average day?

<1>4 or fewer

<2>5-10

<3>11-20

<4>21-30

<5>31+

<6>Don’t know

[Q10] Which, if any, of the following statements BEST applies to you, when it comes to drinking alcohol?

<1>I drink alcohol every day

<2>I drink alcohol but I don't drink alcohol every day

<3>I drink alcohol but only on special occasions (e.g. New Year)

<4>I used to drink alcohol but I have given up now

<5>I have never drunk alcohol

<6>Prefer not to say

##If selected 1, 2 or 3 in Q10

[Q10a] How many units of alcohol have you consumed in the last week? As a guide, a large glass of wine is around 3 units, a pint of lager around 2.5 units and 25ml of 40% spirit is 1 unit.

<1>Under 1

<2>1-10

<3>11-20

<4>21-35

<5>35-50

<6>Over 50

<7>Don’t know

##If selected 1, 2 or 3 in Q10

[Q0b] How many units of alcohol do you typically consume in a week? As a guide, a large glass of wine is around 3 units, a pint of lager around 2.5 units and 25ml of 40% spirit is 1 unit.

<1>Under 1

<2>1-10

<3>11-20

<4>21-35

<5>35-50

<6>Over 50

<7>Don’t know

[Q11] How many sweet, high-energy snacks - e.g. a portion of biscuits or sweets - do you eat on average? If you are unsure, please estimate.

<1>Less than once a week

<2>1 a week

<3>2-3 a week

<4>4-6 a week

<5>1 a day

<6>2 a day

<7>3 a day

<8>4 or more a day

<9>Don’t know

[Q12] How many salty, high-energy snacks - e.g. a portion of crisps or salted nuts - do you eat on average? If you are unsure, please estimate.

<1>Less than once a week

<2>1 a week

<3>2-3 a week

<4>4-6 a week

<5>1 a day

<6>2 a day

<7>3 a day

<8>4 or more a day

<9>Don’t know

[Q13] How many portions of fatty or fried foods - e.g. a slice of pizza or chips - do you eat on average? If you are unsure, please estimate.

<1>Less than once a week

<2>1 a week

<3>2-3 a week

<4>4-6 a week

<5>1 a day

<6>2 a day

<7>3 a day

<8>4 or more a day

<9>Don’t know

Table 1 – policy/behaviour wording for questions 1-5

| Condition | Description | Behaviour X1  The new policy will reduce… | Behaviour X1  The new policy will help solve England’s problem with… |
| --- | --- | --- | --- |
| 1 | Control, Tax, Alcohol | …the number of people who drink in ways that harm their health. | …alcohol. |
| 2 | Asserting, Tax, Alcohol | …the number of people who drink in ways that harm their health. | …alcohol. |
| 3 | Asserting + quantifying, Tax, Alcohol | …the number of people who drink in ways that harm their health. | …alcohol. |
| 4 | Control, Size, Alcohol | …the number of people who drink in ways that harm their health. | …alcohol. |
| 5 | Asserting, Size, Alcohol | …the number of people who drink in ways that harm their health. | …alcohol. |
| 6 | Asserting + quantifying, Size, Alcohol | …the number of people who drink in ways that harm their health. | …alcohol. |
| 7 | Control, Availability, Alcohol | …the number of people who drink in ways that harm their health. | …alcohol. |
| 8 | Asserting, Availability, Alcohol | …the number of people who drink in ways that harm their health. | …alcohol. |
| 9 | Asserting + quantifying, Availability, Alcohol | …the number of people who drink in ways that harm their health. | …alcohol. |
| 10 | Control, Labelling, Alcohol | …the number of people who drink in ways that harm their health. | …alcohol. |
| 11 | Asserting, Labelling, Alcohol | …the number of people who drink in ways that harm their health. | …alcohol. |
| 12 | Asserting + quantifying, Labelling, Alcohol | …the number of people who drink in ways that harm their health. | …alcohol. |
| 13 | Control, Tax, Tobacco | …the number of people who smoke. | …smoking. |
| 14 | Asserting, Tax, Tobacco | …the number of people who smoke. | …smoking. |
| 15 | Asserting + quantifying, Tax, Tobacco | …the number of people who smoke. | …smoking. |
| 16 | Control, Size, Tobacco | …the number of people who smoke. | …smoking. |
| 17 | Asserting, Size, Tobacco | …the number of people who smoke. | …smoking. |
| 18 | Asserting + quantifying, Size, Tobacco | …the number of people who smoke. | …smoking. |
| 19 | Control, Availability, Tobacco | …the number of people who smoke. | …smoking. |
| 20 | Asserting, Availability, Tobacco | …the number of people who smoke. | …smoking. |
| 21 | Asserting + quantifying, Availability, Tobacco | …the number of people who smoke. | …smoking. |
| 22 | Control, Labelling, Tobacco | …the number of people who smoke. | …smoking. |
| 23 | Asserting, Labelling, Tobacco | …the number of people who smoke. | …smoking. |
| 24 | Asserting + quantifying, Labelling, Tobacco | …the number of people who smoke. | …smoking. |
| 25 | Control, Tax, Food | …the number of people who are overweight or obese. | …obesity. |
| 26 | Asserting, Tax, Food | …the number of people who are overweight or obese. | …obesity. |
| 27 | Asserting + quantifying, Tax, Food | …the number of people who are overweight or obese. | …obesity. |
| 28 | Control, Size, Food | …the number of people who are overweight or obese. | …obesity. |
| 29 | Asserting, Size, Food | …the number of people who are overweight or obese. | …obesity. |
| 30 | Asserting + quantifying, Size, Food | …the number of people who are overweight or obese. | …obesity. |
| 31 | Control, Availability, Food | …the number of people who are overweight or obese. | …obesity. |
| 32 | Asserting, Availability, Food | …the number of people who are overweight or obese. | …obesity. |
| 33 | Asserting + quantifying, Availability, Food | …the number of people who are overweight or obese. | …obesity. |
| 34 | Control, Labelling, Food | …the number of people who are overweight or obese. | …obesity. |
| 35 | Asserting, Labelling, Food | …the number of people who are overweight or obese. | …obesity. |
| 36 | Asserting + quantifying, Labelling, Food | …the number of people who are overweight or obese. | …obesity. |
